# A Comprehensive Screening of the Interactors of Areca Palm Necrotic Ringspot Virus (ANRSV) HCPro2 Highlights the Proviral Roles of eIF4A and PGK in Viral Infection

**DOI:** 10.3390/plants14111673

**Published:** 2025-05-30

**Authors:** Li Qin, Peilan Liu, Wentao Shen, Zhaoji Dai, Hongguang Cui

**Affiliations:** 1 Key Laboratory of Green Prevention and Control of Tropical Plant Diseases and Pests (Ministry of Education), School of Tropical Agriculture and Forestry, Hainan University, Haikou 570228, China; qinli0820@126.com (L.Q.); liupeilan0614@163.com (P.L.); zhaoji.dai@hainanu.edu.cn (Z.D.); 2 Institute of Tropical Bioscience and Biotechnology, Chinese Academy of Tropical Agricultural Sciences, Haikou 571101, China; shenwentao@itbb.org.cn

**Keywords:** *Potyviridae*, leader protease, cysteine protease, eukaryotic initiation factor 4A, phosphoglycerate kinase

## Abstract

The areca palm (*Areca catechu* L.), a medicinal tropical crop, hosts three novel viruses, areca palm necrotic ringspot virus (ANRSV), areca palm necrotic spindle-spot virus (ANSSV), and ANRSV2, which form a new genus *Arepavirus* in the family *Potyviridae*. Both viruses feature a unique tandem leader protease arrangement (HCPro1-HCPro2). To elucidate HCPro2’s role, this study identified its interaction partners in infected cells using affinity purification coupled with liquid chromatography-tandem mass spectrometry, a yeast two-hybrid system, and co-immunoprecipitation. Thirteen host proteins and five viral factors (HCPro1, 6K2, VPg, NIa-Pro, NIb) were validated as HCPro2 interactors. Among the host proteins interacting with HCPro2, the expression of five genes (*NbeIF4A*, *NbSAMS1α*, *NbTEF1α*, *NbUEP1,* and *NbRan2*) was upregulated under the condition of viral infection, while the expression of another five genes (*NbpsbS1*, *NbPGK*, *NbchIP*, *NbClpC1A*, and *NbCysPrx*) was downregulated. Functional assays showed that silencing *NbeIF4A* or *NbPGK* significantly reduced viral accumulation *in Nicotiana benthamiana*. These findings reveal HCPro2’s network of virus-host interaction, highlighting its critical role in viral pathogenesis. Further exploration of these interactions may clarify the evolutionary significance of tandem leader proteases and inform novel plant antiviral strategies.

## 1. Introduction

Members in the family *Potyviridae* (potyvirids) represent the largest taxonomic group of plant-infecting RNA viruses, including many notorious viruses such as potato virus Y and plum pox virus (PPV). With the exception of those in the genus *Bymovirus*, all potyvirids possess monopartite single-stranded positive-sense RNA (+ssRNA) genomes (8.2~11.5 kb) encapsidated in filamentous particles measuring 650~950 nm in length and 11~20 nm in diameter [1,2,3]. These genomes feature two overlapping open reading frames (ORFs): a primary genome-spanning ORF, and a short embedded ORF (PIPO) in P3 cistron [1,4]. A conserved mechanism mediated by viral RNA polymerase NIb slippage at the conserved “G_1-2_A_6-7_” motif in the P3 sequence during replication generates translational frame-shift variants containing PIPO in frame with the P3 5′-terminus and its upstream sequence [5,6,7,8]. Along with viral genome translation, two resulting polyproteins (large and small) are immediately processed by virus-encoded proteases into 10~12 mature viral units. The 5′-terminal regions of potyvirid +ssRNA genomes encode two leader proteases—a chymotrypsin-like serine protease (P1) and a cysteine protease (HCPro)—which exhibit remarkable sequence divergence and arrangement across genera or even species. To date, five distinct arrangement patterns have been documented: P1-HCPro tandem, single P1, P1a-P1b tandem, single HCPro, and HCPro1-HCPro2 tandem [7].

Based on phylogenetic analysis, potyvirid HCPro proteins are categorized into three functional groups [9,10]. Group I is comprised of HCPro proteins from most potyvirids, which share a similar size (~450 aa) and retain canonical RNA silencing suppression (RSS) activity [11,12]. HCPro proteins from viruses in the genus *Potyvirus* are well-studied multifunctional effectors [7,13], which are involved in aphid transmission, polyprotein processing, RSS, viral genome translation, and particle yield [14,15,16]. Group II are smaller variants (383~470 aa) lacking RSS activity, with this function compensated by upstream P1 proteins [17,18]. Group III are minimized forms (230~301 aa) with limited characterization. The single HCPro of alpinia oxyphylla mosaic virus suppresses RNA silencing by interfering with double-stranded RNA synthesis [10]. An HCPro homolog (namely P1) in barley yellow mosaic virus also exerts the RSS activity and is indispensable for viral systemic infection [19,20].

Previously, we characterized two novel viruses: areca palm necrotic ringspot virus (ANRSV) and areca palm necrotic spindle-spot virus (ANSSV). In nature, both viruses infect areca palm (*Areca catechu* L.), causing severe foliage chlorosis and necrosis. They are closely related and form a new genus *Arepavirus* in the family *Potyviridae* [1,21,22]. Recently, a third member in the genus, namely areca palm necrotic ringspot virus 2 (ANRSV2), which shares 68.6–68.8% nt identity with ANRSV, was identified from areca palm in India [23]. A distinct arrangement of leader proteases, HCPro1 and HCPro2 in tandem (HCPro1-HCPro2), is a hallmark of the three viruses [9,21,22]. Both HCPro1 and HCPro2 are classified into Group III HCPro proteins [9]. Our recent studies revealed that (i) HCPro1 is dispensable for viral infection; and (ii) HCPro2 exerts RSS activity, but also aids viral intercellular trafficking [9,24].

In a previous study by our group, a variety of host and virus-encoded proteins that are physically associated with HCPro2 in ANRSV infection were identified by affinity purification coupled with liquid chromatography-tandem mass spectrometry (LC-MS/MS). One of them, the Rubisco small unit (RbCS), was experimentally proven to assist viral intercellular movement by serving as the scaffold protein to mediate the interactions among three movement-related proteins: HCPro2, CI, and CP [24]. Nevertheless, it is unknown whether HCPro2 has other interactors. In this study, we aim to screen and identify HCPro2-interacting host and virus proteins. We demonstrate that 13 host proteins and 5 viral factors have interactions with HCPro2. The expression of 10 host genes is changed in response to viral infection. Two tested genes, *NbeIF4A* and *NbPGK*, play crucial roles in viral infection. The obtained data facilitate the understanding of the interactive network of HCPro2 in plant cells and provide a basis for insights into novel functions of Group III HCPro proteins in viral infection.

## 2. Results

### 2.1. Both HCPro1 and HCPro2 Were Immuno-Detected in ANRSV-Infected Cells

**Figure 1:**
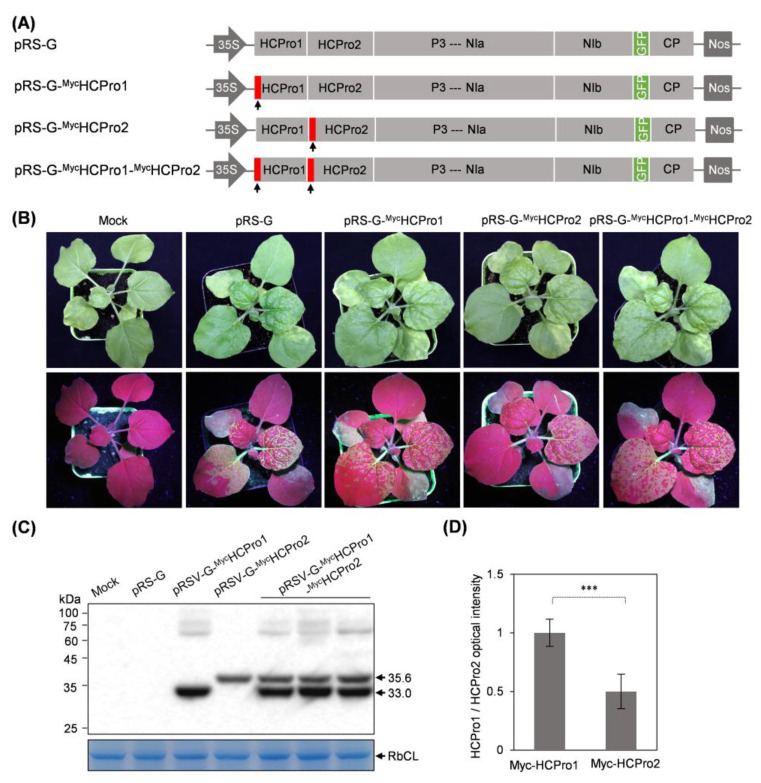
Immunoblot detection of HCPro1 and HCPro2 during viral infection. (**A**) Schematic diagrams of the indicated virus clones. The narrow red boxes by the black arrows denote a Myc-tag. (**B**) Infectivity test of virus clones in *N. benthamiana*. Representative plants were photographed under daylight (upper) and UV light (lower) at 10 days post-inoculation (dpi). Mock, empty vector control. (**C**) Immunoblot detection of Myc-HCPro1 and Myc-HCPro2 in the top new leaves of inoculated plants at 10 dpi. The black arrows indicate the bands corresponding to Myc-HCPro1 (~33.0 kDa) and Myc-HCPro2 (~35.6 kDa). A gel stained with Coomassie blue showing that Rubisco large subunit (RbCL) serves as a loading control. (**D**) Quantitative analysis of the signal intensity of the bands corresponding to Myc-HCPro1 and Myc-HCPro2 in panel C. The values are presented as mean ± standard deviation (SD) (*n* = 3). The mean value for Myc-HCPro1 was designated as 1.0 to normalize the data. Statistically significant differences, determined by an unpaired two-tailed Student’s *t*-test, are indicated by asterisks: *** *p* < 0.001.

To examine the expression status of HCPro1 and HCPro2 in the context of viral infection, two recombinant virus clones, termed pRS-G-MycHCPro1 and pRS-G-MycHCPro2, in which a Myc-tag was fused to the N-terminus of either HCPro1, or HCPro2, respectively, were developed (Figure 1A). Infectivity tests in *N. benthamiana* showed that all plants inoculated with pRS-G, pRS-G-MycHCPro1, or pRS-G-MycHCPro2 (six plants/clone) displayed vein-clearing symptoms and strong green fluorescence signals in the top new leaves (Figure 1B), an indication of viral systemic infection. Western blot detected the expression of Myc-HCPro1 (~33.0 kDa) or Myc-HCPro2 (~35.6 kDa) in diseased leaves (Figure 1C). Subsequently, we developed another virus clone pRS-G-MycHCPro1-MycHCPro2, for which a Myc-tag was fused with the N-termini of both HCPro1 and HCPro2, in order to compare the abundance of HCPro1 and HCPro2 in virus-infected cells (Figure 1A). Similar to pRS-G-MycHCPro1 and pRS-G-MycHCPro2, pRS-G-MycHCPro1-MycHCPro2 is highly infectious in *N. benthamiana* (Figure 1B). Immunoblot analysis showed that Myc-HCPro1 accumulates at a higher level than Myc-HCPro2 (Figure 1C,D), suggesting that HCPro2 might undergo cellular degradation. Given that HCPro2 but not HCPro1 is indispensable for the successful infection of ANRSV [24], this study is committed to the screening and identification of HCPro2 interactors.

### 2.2. Thirteen Host Proteins Are Interactive with HCPro2

A total of 52 host proteins were co-purified with HCPro2 by streptavidin purification and LC-MS/MS during viral infection in *N. benthamiana* [24]. To verify whether these proteins interact with HCPro2, we selected 13 candidate proteins with the LC-MS/MS scores above 25 for further analysis: chloroplast photosystem II 22 kDa component (NbpsbS1), S-adenosyl homocysteine hydrolase (NbSAHH), ubiquitin extension protein 1 (NbUEP1), small GTPase Ran2 (NbRan2), 2-Cys peroxiredoxin (NbCysPrs), alcohol dehydrogenase (NbADH), S-adenosylmethionine synthetase (NbSAMS1α), eukaryotic initiation factor 4A (NbeIF4A), translation elongation factor 1 alpha (NbTEF1α), geranylgeranyl reductase (NbchIP), phosphoglycerate kinase (NbPGK), ATP synthase F1 subunit 1 (Nbatp-A), and chloroplast ATP-dependent Clp protease chaperone protein (NbClpC1A). The coding sequences of these genes were amplified from *N. benthamiana* with corresponding primer sets (Figure S1 and Table S1), followed by integration into Gateway entry vectors.

**Figure 2:**
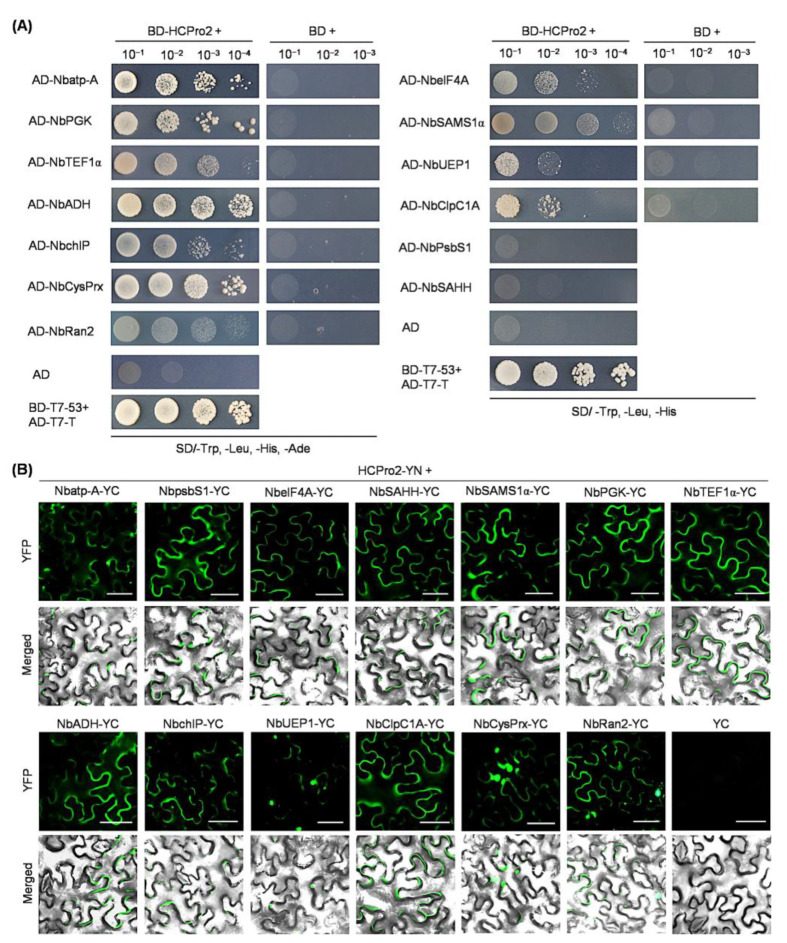
Y2H and BiFC tests of the interactions of HCPro2 with 13 candidate host proteins. (**A**) Y2H tests of the interactions between HCPro2 and host proteins. The indicated pair of proteins was co-transformed into yeast-competent cells. The cells were serially diluted by 10-fold, and cultured on solid mediums of SD/-Trp, -Leu, -His, or SD/-Trp, -Leu, -His, -Ade at 28 °C for four to six days. The co-transformation of two plasmids expressing AD-T7-T and BD-T7-53 served as the positive control. (**B**) BiFC tests the interactions between HCPro2 and host proteins. Each of the YC-fused host proteins, together with HCPro2-YN, were co-expressed in *N. benthamiana* leaves. The co-expression of HCPro2-YN/YC served as the parallel control. The YFP signals (shown in green) were observed at 72 hpi. Bars, 50 μm.

For the test by yeast two-hybrid (Y2H), these sequences were individually transferred into pGADT7-DEST for the expression of fused proteins with the GAL4 AD domain. Each of these constructs, together with the one expressing BD-HCPro2 [24], were co-transformed into yeast cells. As an alternative, these sequences were engineered into pGBKT7-DEST for the expression of fused proteins with the GAL4 BD domain; each of them, along with AD-HCPro2-expressing plasmid [24], was co-delivered into yeast cells. The Y2H results showed that all tested host proteins can interact with HCPro2 (Figure 2A and Figure S2).

Furthermore, bimolecular fluorescence complementation (BiFC) was adopted to verify these interactions *in planta.* The coding sequences of the 13 genes were transferred into pEarleyGate202-YC for the expression of fused proteins with the YC part of the YFP. Each of the YC-fused proteins in combination with HCPro2-YN (HCPro2 fused with the YN part of the YFP) were co-expressed in *N. benthamiana* leaves. At 72 h post-inoculation (hpi), obvious fluorescence signals were observed from all co-inoculated leaf patches under a fluorescence microscope (Figure 2B). In contrast, fluorescence signals were not distinguished from the control samples co-expressing HCPro2-YN/YC or YC-fused protein/YN (Figure 2B and Figure S3). These results demonstrate that all 13 candidate proteins interact with HCPro2 *in planta.*

### 2.3. Five Viral Factors (HCPro1, 6K2, VPg, NIa-Pro, and NIb) Interact with HCPro2 in Planta

**Figure 3:**
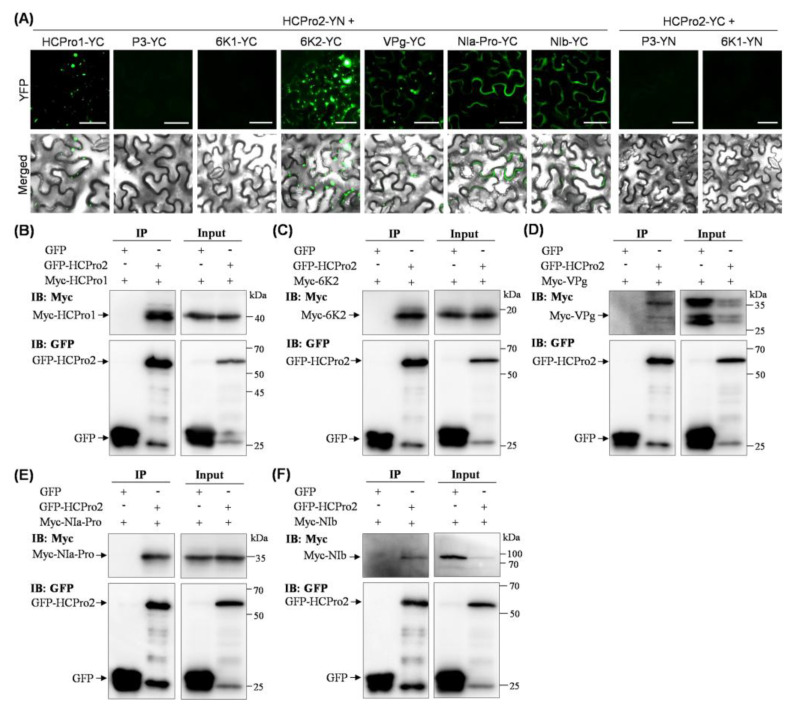
BiFC and Co-IP tests of the interactions between HCPro2 and viral factors. (**A**) BiFC tests of the interactions between HCPro2 and viral factors. The pairs of proteins were co-expressed in *N. benthamiana* leaves. The fluorescence signals were observed at 72 hpi. Bars, 50 μm. (**B**–**F**) Co-IP tests of the interactions between HCPro2 and viral proteins. The fused Myc-HCPro1 (**B**), Myc-6K2 (**C**), Myc-VPg (**D**), Myc-NIa-Pro (**E**), or Myc-NIb (**F**), together with GFP-HCPro2, were co-expressed in *N. benthamiana* leaves. The co-infiltrated leaf patches were collected at 72 hpi for immunoprecipitation by using GFP-Trap Agarose. The co-expression of the indicated proteins with GFP served as parallel controls. Total protein extracts prior to (Input) and after immunoprecipitation (IP) were analyzed by immunoblot using anti-Myc and anti-GFP antibodies.

When tested by Y2H, ANRSV HCPro2 has no interaction with any other viral protein, whereas it interacts with CI and CP *in planta* [24]. Therefore, we determined whether HCPro2 interacts with the remaining viral proteins (HCPro1, P3, 6K1, 6K2, VPg, NIa-Pro, and NIb) *in planta* by BiFC and co-immunoprecipiation (Co-IP) assays. Their coding sequences were individually engineered into pEarleyGate202-YC. Each of the YC-fused proteins, along with HCPro2-YN, were co-expressed in *N. benthamiana* leaves. At 72 hpi, obvious fluorescence signals were observed in leaf patches co-expressing HCPro2-YN/HCPro1-YC, HCPro2-YN/6K2-YC, HCPro2-YN/VPg-YC, HCPro2-YN/NIa-Pro-YC, or HCPro2-YN/NIb-YC (Figure 3A and Figure S4), an indication of the interactions of HCPro2 with HCPro1, 6K2, VPg, NIa-Pro and NIb. In contrast, no interaction was detected between HCPro2 and P3 or 6K1 (Figure 3A and Figure S4). Furthermore, Co-IP assays were conducted to confirm these interactions. We developed five T-DNA constructs for the transient expression of 4 × Myc-tagged HCPro1 (Myc-HCPro1), 6K2 (Myc-6K2), VPg (Myc-VPg), NIa-Pro (Myc-NIa-Pro), and NIb (Myc-NIb). Each of the fused proteins, together with GFP-HCPro2 or GFP (the control), were co-expressed in *N. benthamiana* leaves. At 72 hpi, the co-inoculated leaf patches were collected, followed by co-immunoprecipitation with GFP-Trap Agarose. The results showed that all five viral proteins were co-immunoprecipitated with GFP-HCPro2, but not with GFP alone in the control samples (Figure 3B–F). These results further support the interactions of HCPro2 with HCPro1, 6K2, VPg, NIa-Pro, or NIb. Taken together, in combination with our previous study [24], a total of seven viral factors can interact with HCPro2 *in planta*, and they are HCPro1, CI, 6K2, VPg, NIa-Pro, NIb, and CP.

### 2.4. Expression of Ten Genes Encoding HCPro2-Interacting Proteins Is Changed upon Viral Infection

**Figure 4:**
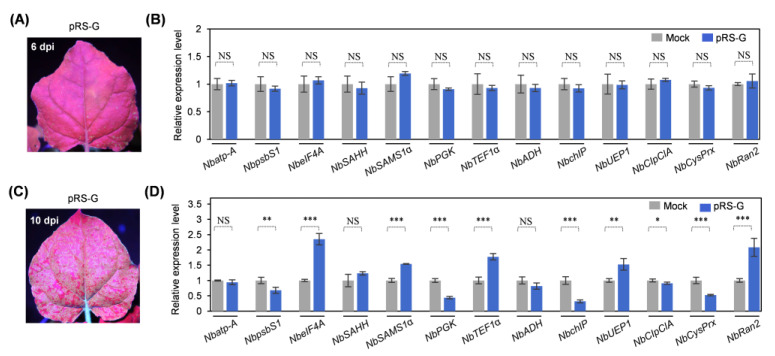
Real-time RT-qPCR analysis of the alternations in mRNA levels of 13 host genes in response to viral infection. (**A**,**C**) Green fluorescence distribution in newly-expanded leaves of representative inoculated plants. The photographs were taken in a dark room under UV light at 6 dpi (**A**) and 10 dpi (**C**). (**B**,**D**) Real-time RT-qPCR analysis of the transcript abundance of indicated genes. The newly-expanded leaves of inoculated plants were sampled at 6 dpi (**B**) and 10 dpi (**D**) for the test. The expression level of *NbActin* was determined to normalize the data. The SD values were calculated from four biological replicates. Statistically significant differences, determined by an unpaired two-tailed Student’s *t*-test, are indicated by asterisks: * 0.01 < *p* < 0.05; ** 0.001 < *p* < 0.01; *** *p* < 0.001; NS, 0.05 < *p*.

Next, we examined possible changes in the expression levels of these 13 host genes in response to viral infection. Both pRS-G and pCB301 (empty vector) were respectively inoculated into *N. benthamiana* plants (10 plants per construct). The newly-expanded leaves of the inoculated plants were sampled for the analysis at two different time points: (i) 6 dpi, at which the newly-expanded leaves started to manifest green fluorescence signals (Figure 4A), indicating the onset of viral systemic infection; (ii) 10 dpi, at which the newly-expanded leaves were systemically infected (Figure 4C). Real-time RT-qPCR analysis revealed that the expression levels of all the tested genes have no significant differences at 6 dpi (Figure 4B). However, 10 are responsive to viral infection at 10 dpi: the transcript abundance of five genes (*NbpsbS1*, *NbPGK*, *NbchIP*, *NbClpC1A*, and *NbCysPrx*) were significantly downregulated, whereas another five genes (*NbeIF4A*, *NbSAMS1α*, *NbTEF1α*, *NbUEP1*, and *NbRan2*) were upregulated (Figure 4D). The remaining three genes—*Nbatp-A*, *NbSAHH1α*, and *NbADH*—did not show significant expression changes at either time point.

### 2.5. Silencing of NbeIF4A or NbPGK via TRV-Based VIGS Abolishes or Significantly Impairs Viral Systemic Infection

**Figure 5:**
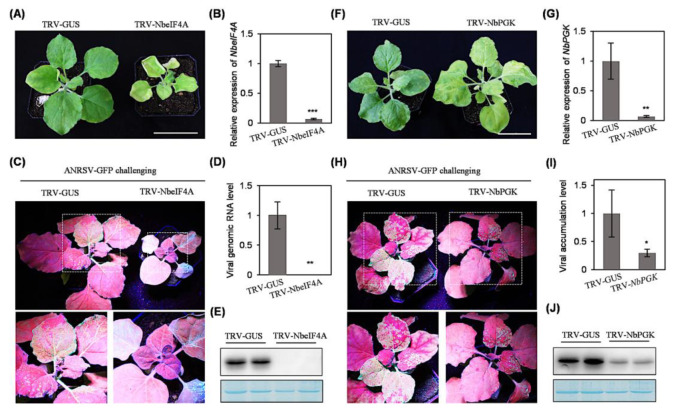
The effects of silencing *NbeIF4A* or *NbPGK* on viral systemic infection in *N. benthamiana*. (**A**,**F**) The phenotypes of silencing *NbeIF4A* or *NbPGK* in *N. benthamiana*. An agrobacterial culture harboring pTRV1, together with that of pTRV2-NbeIF4A (**A**), pTRV2-NbPGK (**F**), or pTRV-GUS (as the parallel control), were co-infiltrated into *N. benthamiana* seedlings (8 plants per treatment). The representative plants were photographed at 12 dpi. Bars, 5 cm. (**B**,**G**) Real-time RT-qPCR analysis of the expression level of *NbeIF4A* or *NbPGK* at 12 dpi. The transcript abundance of *NbActin* was determined to normalize the data. The SD values were calculated from four biological replicates. Statistically significant differences, determined by an unpaired two-tailed Student’s *t*-test, are indicated by asterisks: ** 0.001 < *p* < 0.01, *** *p* < 0.001. (**C**,**H**) Silencing of *NbeIF4A* or *NbPGK* abolishes or significantly restricts viral systemic infection. The *N. benthamiana*, pre-inoculated with TRV-GUS, TRV-NbeIF4A, or TRV-NbPGK at 12 dpi, were challenged with ANRSV-GFP via sap rub-inoculation. The representative plants were photographed in a dark room under UV light at 12 days post-challenging inoculation (dpci). The close view of leaf regions indicated by dashed boxes is shown. (**D**,**I**) Real-time RT-qPCR analysis of viral genomic RNA accumulation. The upper non-inoculated leaves were collected at 12 dpci. The transcript abundance of *NbActin* was determined to normalize the data. The SD values were calculated from four biological replicates. * 0.01 < *p* < 0.05; ** 0.001 < *p* < 0.01. (**E**,**J**) Immunoblot analysis of GFP accumulation at 12 dpci. A gel stained by Coomassie blue showing that RbCL serves as a loading control.

We selected two candidate host proteins—NbeIF4A and NbPGK—that interact with HCPro2 to evaluate their effects on viral infection, mainly considering that *NbeIF4A* and *NbPGK*/*NbchIP* are the most upregulated and downregulated genes in response to viral infection (Figure 4D). TRV-based VIGS [25] was employed to knock down their expression in *N. benthamiana*. At 12 dpi, *NbeIF4A*-silenced plants manifested extreme dwarfism phenotype, while *NbPGK*-silenced ones exhibited foliage yellowing (Figure 5A,F). These symptoms were not observed in control plants infected with TRV-GUS (Figure 5A,F). Real-time RT-qPCR confirmed a significant decrease in transcript abundance of *NbeIF4A* and *NbPGK* in corresponding plants (Figure 5B,G). At this time point, the newly expanded leaves of inoculated plants were subjected to challenging inoculation with ANRSV-GFP via sap rub-inoculation. Twelve days later, the newly emerging leaves of all the plants inoculated with TRV-GUS showed strong fluorescence signals, indicating a systemic infection by ANRSV-GFP. However, no GFP signals were observed in the top new leaves of *NbeIF4A*-silenced plants (Figure 5C). Real-time RT-qPCR and immunoblot analysis confirmed that ANRSV-GFP failed to systemically infect *NbeIF4A*-silenced plants (Figure 5D,E). In comparison with the plants treated with TRV-GUS, a lower level of green fluorescence was observed in the upper leaves of *NbPGK*-silenced plants (Figure 5H). Real-time RT-qPCR and immunoblot analysis confirmed that silencing of *NbPGK* significantly impairs viral systemic infection (Figure 5I,J).

### 2.6. Transient Silencing of NbeIF4A or NbPGK in Local Leaves Through Expressing a Hairpin Structure Significantly Attenuates Viral Infectivity

**Figure 6:**
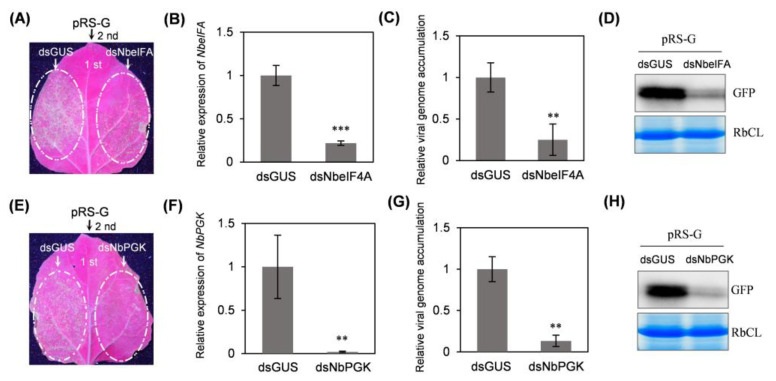
Transient silencing of *NbeIF4A* or *NbPGK* significantly attenuates viral infectivity in local leaves. (**A**,**E**) Infectivity test of pRS-G in *NbeIF4A*- or *NbPGK*-silenced leaves. The representative leaves were photographed in a dark room under UV light at 84 hpi with pRS-G. (**B**,**F**) Real-time RT-qPCR analysis of the expression level of *NbeIF4A* (**B**) or *NbPGK* (**F**) at 84 hpi with pRS-G. The transcript abundance of *NbActin* was determined to normalize the data. The SD values were calculated from four biological replicates. Statistically significant differences, determined by an unpaired two-tailed Student’s *t*-test, are indicated by asterisks: ** 0.001 < *p* < 0.01; *** *p* < 0.001. (**C**,**G**) Real-time RT-qPCR analysis of viral genomic RNA accumulation at 84 hpi with pRS-G. (**D**,**H**) Immunoblot analysis of GFP accumulation at 84 hpi with pRS-G.

Silencing of *NbeIF4A* or *NbPGK* via TRV-based VIGS leads to severe physiological abnormality (Figure 5). Thus, we performed transient silencing of *NbeIF4A* and *NbPGK* via the expression of a hairpin structure to test viral infectivity in local leaves. For this, a fragment of *NbeIF4A* or *NbPGK* (~300 bp) was cloned into a hairpin RNAi plasmid (p2300s-intron). The construct p2300s-intron-dsGUS harboring a fragment of GUS [25] was included as the parallel control. These constructs were inoculated into fully expanded leaves of *N. benthamiana* plants at an OD_600_ of 0.3 per construct (Figure 6A,E). At 24 hpi, an agrobacterial culture carrying pRS-G (OD_600_ = 0.3) was re-infiltrated into the leaf patches (Figure 6A,E). Eighty-four hours later, real-time RT-qPCR confirmed that the transcript abundance of *NbeIF4A* or *NbPGK* was significantly reduced (Figure 6B,F). At this time point, obvious green fluorescence signals, an indication of ANRSV-GFP infection, were observed in leaf patches treated with dsGUS/pRS-G, whereas *NbeIF4A*- or *NbPGK*-silenced leaf patches manifested relatively weak fluorescence (Figure 6A,E). Consistently, real-time RT-qPCR and immunoblot analysis revealed that the accumulation levels of viral genomic RNA and protein were significantly decreased in *NbeIF4A*- or *NbPGK*-silenced leaf patches (Figure 6C,D,G,H). Together, the above data indicate that transient silencing of *NbeIF4A* or *NbPGK* in local leaves significantly attenuates viral infectivity.

## 3. Discussion

HCPro is a highly variable potyvirid factor. Dissecting its interactions with host and virus-encoded proteins to reveal its biological functions would provide insight into its roles in viral host adaption and host range expansion. Although Group I HCPro proteins having multifaceted roles in viral infection are well studied, the biological functions of those in Group III are far from understood. This study demonstrates that ANRSV HCPro2 can interact with a variety of host and viral proteins. Two tested proteins—NbeIF4A and NbPGK—positively regulate viral infection. Investigating the biological relevance underpinning these interactions might provide a basis for developing novel antiviral strategies.

eIF4A is a member of the DEAD-box RNA helicase protein family that typically functions in unwinding the mRNA structure for ribosome recruitment during translation initiation [26]. In cases of several plant viruses, such as tomato bushy stunt virus and brome mosaic virus (BMV), it was proven that eIF4A can stimulate viral genome translation and/or replication via binding *cis*-acting element in 5′- or 3′-UTR in viral genome [27]. In addition, eIF4A is proven to be a negative regulator of cellular autophagy, and involved in autophagy-mediated antiviral pathways [28,29]. A recent study revealed that NbeIF4A interacts with a variety of virus-encoded movement proteins of the 30K family [30], although the biological relevance underlying these interactions is not elucidated. Interestingly, ANRSV HCPro2, besides having RSS activity, also aids viral intercellular trafficking [9,24]. This study validated that NbeIF4A, an HCPro2’ interactor, positively regulates ANRSV infection. We attempt to speculate that NbeIF4A interacts with HCPro2 to facilitate viral genome translation, replication, and/or intercellular movement. To test this hypothesis, it is needed to map protein regions involved in the interaction between NbeIF4A and HCPro2, and examine the effects on viral genome translation/replication via destroying the interaction site. In addition, the binding affinity of NbeIF4A or HCPro2 with 5′- and 3′-UTRs should be examined. Nevertheless, this study represents the first report of the interaction of NbeIF4A with HCPro, and its proviral role in potyvirid infection.

NbPGK was shown to interact with the viral genome of BMV and is required for robust viral multiplication [31]. For two potyviruses (e.g., watermelon mosaic virus and plum pox virus), the host factor PGK was mapped to confer recessive resistance [32,33]. However, the underlying action mechanisms are unknown. Once *NbPGK* was silenced, a similar resistance was observed for ANRSV. In addition, its interaction with HCPro2 was corroborated. Altogether, the PGK is a host factor, which supports the infections by a variety of plant viruses. Curiously, *NbPGK* is downregulated during ANRSV infection. We propose that *NbPGK* downregulation likely results from host-mediated regulation of its expression to confer host defense response to restrict viral infection. Nevertheless, it remains to be investigated whether NbPGK promotes viral infection via an interaction of HCPro2. As stated in the Introduction, Group I HCPro proteins are multifunctional effectors. Among these functions, the RSS is well elucidated. They exert RSS activity by interfering with different stages of the RNAi pathway [7,13]. The majority of HCPro proteins sequester virus-derived small interfering RNA (vsiRNA) to prevent their entry into silencing effector complexes [18,34,35]. Besides sequestering vsiRNAs, other mechanisms are also documented: (i) The inhibition of siRNA methylation. Potato virus A (PVA) HCPro disrupts the methionine cycle via interacting with SAHH and SAMS [36]. In the cases of zucchini yellow mosaic virus and turnip mosaic virus, HCPro interacts with HUA enhancer 1 (HEN1) to inhibit its methyltransferase activity [37,38]. (ii) Interference with the effector protein AGO1 [36,39]. (iii) Disturbance of RDR6-mediated dsRNA amplification [40]. A subset of HCPro proteins in Group III, including ANRSV HCPro2, were experimentally proven to have RSS activity, whereas the action mechanisms have not yet been elucidated. It is noticed that NbSAHH and NbSAMS1α are in the list of HCPro2 interactors. We propose that ANRSV HCPro2, behaving like PVA HCPro, might disrupt the cellular methionine cycle by interacting with NbSAHH and NbSAMS1α to suppress RNA silencing.

A variety of host proteins, besides those mentioned above, have been reported to interact with different HCPro proteins. They interact with translation initiation factor eIF4E or its isoform eIF(iso)4E, likely promoting viral genome translation [41,42,43]. Several chloroplastic proteins are also the interactors, such as ATP synthase CF1 β-subunit, Ferredoxin-5, MinD (a member of ParA ATPase family), and Rubisco [43,44,45,46,47]. Although these interactions usually affect photosynthetic pathways, their biological relevance in viral infection is nearly obscure. For other HCPro interactors, the readers are directed to several excellent reviews [13,35,43]. It is interesting that all these previously reported host proteins are not in the list of ANRSV HCPro2 interactors, likely implying that HCPro2 might have distinct functions in viral infection. Again, dissecting the biological relevance of the interactions of HCPro2 with 13 host proteins would provide insight into new functions of this highly divergent HCPro.

Previous studies reveal that several viral factors interact with different HCPro proteins—P1, CI, VPg, NIa, and CP ([48,49,50,51,52,53,54,55,56]—although the biological significance of these interactions is less elucidated. In combination with our previous study [24], ANRSV HCPro2 can interact with seven viral factors (HCPro1, CI, 6K2, VPg, NIa-Pro, NIb, and CP). Intriguingly, these interactions consistently occur *in planta* rather than in yeast, implying that HCPro2 interacts with these viral factors likely via cellular proteins as the mediators. Indeed, we demonstrated that HCPro2 interacts with either CI or CP by utility of host protein—RbCS—as the scaffold to assemble a movement complex to aid viral intercellular trafficking [24]. Future studies should concentrate on: (i) which host proteins mediate the interactions of HCPro2 with viral factors, and (ii) the roles of the complex HCPro2/host protein/viral factor in viral infection. These will be promising research directions.

## 4. Materials and Methods

### 4.1. Plant Material

*Nicotiana benthamiana* seedlings were maintained in a growth chamber with the parameter setup as 16 h of light at 25 °C and 8 h of darkness at 23 °C (65% relative humidity).

### 4.2. Plasmid Construction

pRS-G, a GFP-tagged infectious cDNA clone of ANRSV isolate ANRSV-ZYZ, was previously developed by our group [57]. Using pRS-G as the backbone, we developed three recombinant virus clones: pRS-G-MycHCPro1, pRS-G-MycHCPro2, and pRS-G-MycHCPro1-MycHCPro2. These clones were produced by a similar strategy. Herein, we presented a detailed description of the creation of pRS-G-MycHCPro1. Two PCRs with the pRS-G as the template were performed using primer sets pCB301-F/RSV-SOE-M1M2-2 and RSV-SOE-M1M2-3/RSV-1-R (Table S1). The obtained PCR products were mixed as the template for overlapping PCR with primer set PCB301-F/RSV-1-R (Table S1), and the resulting amplicon was inserted back into pRS-G by utility of *Pme* I/*Nhe* I sites.

The complete coding sequences of 13 candidate host genes were retrieved from the NCBI GenBank database (*NbatpA*, MN365024; *NbpsbS1*, DQ340567; *NbeIF4A*, JN688263; *NbSAHH*, JQ890096; *NbSAMS1α*, LC008353; *NbPGK*, HQ450764; *NbTEF1α*, PQ008965; *NbchIP*, AJ584638; *NbUEP1*, MG333694; *NbClpC1A*, KJ406176; *NbRan2*, LC422004) or SGN database (*NbADH*, Niben101Scf04906g00013; *NbCysPrx*, Niben101Scf01372g00003) for the design of primers (Table S1). For yeast two-hybrid (Y2H), bimolecular fluorescence complementation (BiFC), and co-immunoprecipitation (Co-IP) assays, the indicated genes were first cloned into pDONR221 to create entry clones by using Gateway cloning technology. The entry clones were transferred into corresponding destination vectors, pGADT7-DEST, pGBKT7-DEST, pEarleygate201-YN, pEarleygate202-YC, and/or pBA-FLAG-4myc-DC [58,59,60]. The construct pCaM-GFP-HCPro2, expressing the fused GFP-HCPro2, was generated previously [24].

For tobacco rattle virus (TRV)-based virus-induced gene silencing (VIGS) [25], an online tool for SGN VIGS (http://vigs.solgenomics.net) (accessed on 25 April 2022) was used to design primer sets (Table S1) amplifying corresponding regions of *NbeIF4A* and *NbPGK*. The obtained amplicons (~300 bp) were integrated into pTRV2 via *BamH* I/*Xho* I sites to generate pTRV2-NbeIF4A and pTRV2-NbPGK. For transient silencing assay, the obtained amplicons by corresponding primer sets (Table S1) were engineered into p2300s-intron to generate p2300s-intron-dsNbeIF4A and p2300s-intron-dsNbPGK. pTRV2-GUS and p2300s-intron-dsGUS were prepared previously [10,24].

### 4.3. Agrobacterium-Mediated Inoculation and Sap Rub-Inoculation

The relevant plasmids were transformed into *A. tumefaciens* (the strain GV3101) competent cells by electroporation. For the infectivity test, agrobacterial cultures harboring virus clones were adjusted to 1.0 of optical density at 600 nm (OD_600_) and infiltrated into *N. benthamiana* seedlings at the 3- or 4-leaf stage. For transient expression, fully-expanded leaves of *N. benthamiana* plants at the 6- to 8-leaf stage were infiltrated by agrobacterial cultures harboring relevant plasmids. For co-infiltration, two agrobacterial cultures were respectively adjusted to OD_600_ of 0.6, and mixed with equal volume.

For TRV-VIGS, *N. benthamiana* seedlings at the 3- to 5-leaf stage were co-infiltrated with agrobacterial cultures harboring pTRV1, together with pTRV2-GUS (TRV-GUS), pTRV2-NbeIF4A, or pTRV2-NbPGK (final OD_600_ = 0.3 per culture). Sap rub-inoculation assay was performed by following a previously described protocol [57]

### 4.4. Y2H

Y2H assay was performed by following the manual—Yeastmaker Yeast Transformation System 2 User Manual (Clontech, Mountain View, CA, USA). Each pair of the indicated plasmids was co-transformed into yeast-competent cells (the strain Y2H Gold). The cultures were subjected to 10-fold serial dilution and then plated onto synthetically defined medium, including leucine and tryptophan dropout medium (SD/-Leu/-Trp), leucine, tryptophan and histidine dropout medium (SD/-Leu/-Trp/-His), and/or leucine, tryptophan, histidine, and adenine dropout medium (SD/-Leu/-Trp/-His/-Ade).

### 4.5. BiFC

The indicated genes were cloned into pEarleyGate201-YN for the expression of fused proteins with the N-terminal half of YFP (YN), and pEarleyGate202-YC for fused proteins with the C-terminal half of YFP (YC). Each pair of YN- and YC-fused proteins was co-expressed in fully expanded leaves of *N. benthamiana* plants. A fluorescence microscope (BX53, OLYMPUS, Shinjuku City, Japan) was employed to monitor YFP fluorescence signals in inoculated leaf patches.

### 4.6. Co-IP

Co-IP assay was performed, essentially as previously described [24]. The GFP-Trap Agarose (ChromoTek, Planegg, Germany) was used to immunoprecipitate GFP or GFP-HCPro2.

### 4.7. Reverse-Transcription Polymerase Chain Reaction (RT-PCR) and Real-TIME RT Quantitative PCR (RT-qPCR)

Total RNAs from *N. benthamiana* leaves were extracted by using TRNzol universal RNA reagent (TIANGEN, Beijing, China), and after digestion with DNase I (Thermo Scientific, Waltham, MA, USA), were used to synthesize the first-stranded cDNAs by using the RevertAid First Strand cDNA Synthesis Kit (Thermo Scientific). The cDNAs were used for PCR reactions with corresponding primer sets (Table S1) by using Phusion DNA Polymerase (Thermo Scientific). For real-time RT-qPCR, a pair of primers RSV-9200F/RSV-9350R [57], targeting the viral CP-coding region, were used to determine viral genome RNA accumulation levels. The other pairs of primers, for the determination of transcript abundance of endogenous genes in *N. benthamiana*, were designed using Primer3Plus (https://www.primer3plus.com/index.html) (accessed on 12 March 2022). The qPCR reactions with SuperReal Premix Plus (TIANGEN) were conducted by using Applied Biosystems QuantStudio 5 (Thermo Scientific). *NbActin* serves as the internal reference gene.

## 5. Conclusions

This study experimentally demonstrated that thirteen host proteins and five viral factors are physically interactive with ANRSV HCPro2. Among them, ten host genes are responsive to viral infection, and two tested genes play proviral roles in viral infection. Understanding the biological relevance underpinning these interactions would disclose novel functions of HCPro2, and inspire new approaches to plant disease management.

## Data Availability

The datasets presented in this study can be found in online repositories. The names of the repository/repositories and accession number(s) can be found in the article or Supplementary Material.

## Supplementary Materials

The following supporting information can be downloaded at: https://www.mdpi.com/article/10.3390/plants14111673/s1. Figure S1: RT-PCR amplification of complete coding sequences of host genes; Figure S2: Y2H test of the interactions of HCPro2 with host proteins; Figure S3: BiFC tests of the interactions between YN and host proteins; Figure S4: BiFC tests of the interactions of YN or YC with the indicated proteins; Table S1: Title primers used in this study.

## Abbreviations

The following abbreviations are used in this manuscript:

ADH	alcohol dehydrogenase
ANRSV	areca palm necrotic ringspot virus
ANSSV	areca palm necrotic spindle-spot virus
atp-A	ATP synthase F1 subunit 1
BiFC	bimolecular fluorescence complementation
BMV	brome mosaic virus
chIP	geranylgeranyl reductase
ClpC1A	chloroplast ATP-dependent Clp protease chaperone protein
Co-IP	co-immunoprecipiation
CysPrs	2-Cys peroxiredoxin
dpi	day post-inoculation
eIF4A	eukaryotic initiation factor 4A
HEN1	HUA enhancer 1
hpi	hours post-inoculation
LC-MS/MS	liquid chromatography-tandem mass spectrometry
OD600	optical density at 600 nm
ORFs	open reading frames
PGK	phosphoglycerate kinase
PPV	plum pox virus
psbS1	chloroplast photosystem II 22 kDa componentry
PVA	potato virus A
Ran2	small GTPase Ran2
RbCS	Rubisco small unit
RT-PCR	reverse-transcription polymerase chain reaction
RT-qPCR	real-time RT quantitative PCR
RSS	RNA silencing suppression
SAHH	S-adenosyl homocysteine hydrolase
SAMS1α	S-adenosylmethionine synthetase
SD/-Leu/-Trp	synthetically defined medium, including leucine and tryptophan dropout medium
SD/-Leu/-Trp/-His	synthetically defined medium, including leucine, tryptophan, and histidine dropout medium
SD/-Leu/-Trp/-His/-Ade	synthetically defined medium, including leucine, tryptophan, histidine, and adenine dropout medium
ssRNA	single-stranded RNA
TEF1α	translation elongation factor 1 alpha
TRV	tobacco rattle virus
UEP1	ubiquitin extension protein 1
VIGS	virus-based virus-induced gene silencing
Y2H	yeast two-hybrid
