# Supplementary material for: A Comprehensive Screening of the Interactors of Areca Palm Necrotic Ringspot Virus (ANRSV) HCPro2 Highlights the Proviral Roles of eIF4A and PGK in Viral Infection
Source: Plants (Basel). 2025 May 30;14(11):1673. doi: 10.3390/plants14111673 (PMC12158156; doi:10.3390/plants14111673)
Supplement: Supplementary file 1 [file plants-14-01673-s001.zip › plants-3629105-supplementary.pdf]

Supplementary Materials

Table S1 Primers used in this study.

Figure S1. RT-PCR amplification of complete coding sequences of host genes. RT-PCR reactions were performed with gene-specific primer sets (Table S1). M1 and M2, D2000 and Maker III DNA ladders (TIANGEN).

Figure S2. Y2H test the interactions of HCPro2 with host proteins. The indicated pairs of constructs were co-transformed into yeast competent cells, which were subjected to 10-fold serial dilutions and cultured on the solid mediums of SD/-Trp, -Leu, -His, and SD/-Trp, -Leu, -His, -Ade at 28˚C for four to six days.

Figure S3. BiFC tests the interactions between YN and host proteins. The indicated pairs of proteins were co-expressed in *N. benthamiana* leaves. The YFP signals (shown in green) were observed at 72 hpi. Bars, 50 μm.

Figure S4. BiFC tests the interactions of YN or YC with the indicated proteins. The yellow fluorescence signals (shown in green) were observed at 72 hpi. Bars, 50 μm.

Table S1

Primer name	Sequence (5' - 3')	Use
pCB301-F*	TACCCGCCAATATATCCTGTC	pRS-G-MycHCPro1, pRS-G-MycHCPro2 and pRS-G-MycHCPro1-MycHCPro2
RSV-1-R*	GCTAGCTTTCCATGTGATTTGTTTTCCTCTCG	
RSV-SOE-M1M2-2	CCATCAGATCTTCTTCAGAGATCAGTTTCTGTTCCATTGTTGCCCCTGCAATTGGTG	
RSV-SOE-M1M2-3	ACAATGGAACAGAAACTGATCTCTGAAGAAGATCTGATGGCTACACCATCGAAGGC	
RSV-SOE-M1M2-4	GCTAGGTCCTCCTCGGAAATTAGCTTTTGCTCGTGTGCACCAAGCAGCCATGTTGC	
RSV-SOE-M1M2-5	GTGCACACGAGCAAAAGCTAATTTCCGAGGAGGACCTAGCACACCAAGATGAGGAA	
attB1-NbatpA-F	GGGGACAAGTTTGTACAAAAAAGCAGGCTTCATGGAACTTTCTCCCCGAGC	Y2H and BiFC (Gateway cloning)
attB2-NbatpA-R	GGGGACCACTTTGTACAAGAAAGCTGGGTCAATAAAAGCTAAAGCACTTTC	
attB1-NbPPO-F	GGGGACAAGTTTGTACAAAAAAGCAGGCTTCATGGCTTCTTCTTCTACGTTAC	
attB2-NbPPO-R	GGGGACCACTTTGTACAAGAAAGCTGGGTCACAATCGACAAGCTTAATCTC	
attB1-NbPGK-F	GGGGACAAGTTTGTACAAAAAAGCAGGCTTCATGGCATCAGCTACAGCTTC	
attB2-NbPGK-R	GGGGACCACTTTGTACAAGAAAGCTGGGTCCACAGCAAAGGGGGCATCTG	
attB1-NbTEF1α-F	GGGGACAAGTTTGTACAAAAAAGCAGGCTTCATGGGTAAAGAGAAGGTTCAC	
attB2-NbTEF1α-R	GGGGACCACTTTGTACAAGAAAGCTGGGTCCTTTTTCTTCTGGGCAGCCTTG	
attB1-NbADH-F	GGGGACAAGTTTGTACAAAAAAGCAGGCTTCATGGCAAAAAACCCAGAAGAAG	
attB2-NbADH-R	GGGGACCACTTTGTACAAGAAAGCTGGGTCAGCAGAGTTGGAATGGTTGC	
attB1-NbChlP-F	GGGGACAAGTTTGTACAAAAAAGCAGGCTTCATGGCTTCCATTGCTCTCAAAAC	
attB2-NbChlP-R	GGGGACCACTTTGTACAAGAAAGCTGGGTCTACACTGAGCTTGTCCATTTC	
attB1-NbPrx-F	GGGGACAAGTTTGTACAAAAAAGCAGGCTTCATGGCTTGCAGTGCTACTTC	
attB2-NbPrx-R	GGGGACCACTTTGTACAAGAAAGCTGGGTCTATGGATGCAAAGTATTCTTTG	
attB1-NbRan2-F	GGGGACAAGTTTGTACAAAAAAGCAGGCTTCATGGCTCTCCCTAACCAAC	
attB2-NbRan2-R	GGGGACCACTTTGTACAAGAAAGCTGGGTCTTCAAAAGCTTCATCATCGTC	
attB1-NbeLF4A-F	GGGGACAAGTTTGTACAAAAAAGCAGGCTTCATGGCTGGCTTAGCACCGGAAG	
attB2-NbeLF4A-R	GGGGACCACTTTGTACAAGAAAGCTGGGTCAAGGAGATCGGCCACATTGGC	
attB1-NbUEP1-F	GGGGACAAGTTTGTACAAAAAAGCAGGCTTCATGCAGATCTTCGTGAAAAC	
attB2-NbUEP1-R	GGGGACCACTTTGTACAAGAAAGCTGGGTCATCGGCACCGGCCTTGTTGT	
attB1-NbClpC1A-F	GGGGACAAGTTTGTACAAAAAAGCAGGCTTCATGGCTAGAGCTTTAGTTCAG	
attB2-NbClpC1A-F	GGGGACCACTTTGTACAAGAAAGCTGGGTCCACAGGGATAGGCTCAGGAG	
attB1-NbPsbS1-F	GGGGACAAGTTTGTACAAAAAAGCAGGCTTCATGGCTCAAACAATGTTGCTG	
attB2-NbPsbS1-F	GGGGACCACTTTGTACAAGAAAGCTGGGTCCTCCTCTTCCTCATCAGTGAC	
attB1-NbSAMS1α-F	GGGGACAAGTTTGTACAAAAAAGCAGGCTTCATGGAAACTTTCTTGTTCAC	
attB2-NbSAMS1α-R	GGGGACCACTTTGTACAAGAAAGCTGGGTCAGCTTTTGGCTTGAGGACCTTG	
TRV-GUS-F*	ATATAGGATCCTCTGGTATCAGCGCGAAGTCT	pTRV2-GUS
TRV-GUS-R*	ATATACTCGAGTAGTTAAAGAAATCATGGAAG	
TRV-NbPGK-F	ATATAGGATCCTTAGTCGGGGCAGTTTCAAAT	pTRV2-NbPGK
TRV-NbPGK-R	ATATACTCGAGGTTTGCATCAGGAGCAAATT	
TRV-NbeIF4A-F	ATATAGGATCCACTGCTGCCACCAAAGATTC	pTRV2-NbeIF4A
TRV-NbeIF4A-R	ATATACTCGAGGCAGATACTGTATGATCACG	
GUS-RNAi-1F*	ATATAGAGCTCTACGTCCTGTAGAAACCCCAAC	p2300s-intron-NbGUS
GUS-RNAi-1R*	ATATAGGATCCATCACTTCCTGATTATTGAC	
GUS-RNAi-2F*	ATATATCTAGAATCACTTCCTGATTATTGAC	

GUS-RNAi-2R*	ATATACTGCAGTACGTCCTGTAGAAACCCCAAC		
NbeIF4A-RNAi-1F	ATATAGAGCTCACTGCTGCCACCAAAGATTC	p2300s-intron-NbeIF4A	
NbeIF4A-RNAi-1R	ATATAGGATCCGCAGATACTGTATGATCACG		
NbeIF4A-RNAi-2F	ATATATCTAGAGCAGATACTGTATGATCACG		
NbeIF4A-RNAi-2R	ATATACTGCAGACTGCTGCCACCAAAGATTC		
NbPGK-RNAi-1F	ATATAGAGCTCTTAGTCGGGGCAGTTTCAAAT	p2300s-intron-NbPGK	
NbPGK-RNAi-1R	ATATAGGATCCGTTTGCATCAGGAGCAAATTTA		
NbPGK-RNAi-2F	ATATATCTAGAGTTTGCATCAGGAGCAAATTTA		
NbPGK-RNAi-2R	ATATACTGCAGTTAGTCGGGGCAGTTTCAAAT		
NbPGK-qPCR-F	ACTTGATGGCCAATGGTGCTA	Efficiency: 110.01%	real-time qPCR
NbPGK-qPCR-R	CTCAGCCTTCACAACCTGGAT		
NbTEF1α-qPCR-F	ATTGGGTCGTTTTGCTGTGAGG	Efficiency: 108.08%	
NbTEF1α-qPCR-R	TTTTTCTTCTGGGCAGCCTTGG		
NbRan2-qPCR-F	TTTGTGGAATCACCCGCACTTG	Efficiency: 108.50%	
NbRan2-qPCR-R	AGCTTCATCATCGTCATCGGGA		
NbeIF4A-qPCR-F	GGAGACATGGACCAGAACACTAG	Efficiency: 110.31%	
NbeIF4A-qPCR-R	GCTGAGTTGGAAGGTCGTAGTTA		
NbClpC1A-qPCR-F	TTGGAAGGGGTAGTGGGTTTGTT	Efficiency: 108.60%	
NbClpC1A-qPCR-R	CCTTCACGCAGCAATCCAAGTAG		
NbPsbS1-qPCR-F	CCCTACCCCTCCTACTGGTCTT	Efficiency: 103.94%	
NbPsbS1-qPCR-R	TCCCAGTTGTGCCAATCTTCCT		
NbSAMA1α-qPCR-F	AACCATCTTCCACCTCAACCC	Efficiency: 108.36%	
NbSAMA1α-qPCR-R	CTGAGAAAGCACCTCCACCAT		
NbADH-qPCR-F	TGTTGAAGTCGTGCCAATGGAC	Efficiency: 109.04%	
NbADH-qPCR-R	AGCAGAGTTGGAATGGTTGCC		
NbPrx-qPCR-F	GCTTTCAACCCTTCACCTTCTCAC	Efficiency: 101.41%	
NbPrx-qPCR-R	CGACGAAGGAGCGAGATGATTTG		
Nbatp-A-qPCR-F	ATCGGTCGAGTGGTCTCAGTTG	Efficiency: 108.16%	
Nbatp-A-qPCR-R	AGGCTATTCCTTTCACACCGCT		
NbUEP1-qPCR-F	ACGTGAAGGCAAAGATCCAGGA	Efficiency: 107.43%	
NbUEP1-qPCR-R	TTCGTCCATCCTCCAGCTGTTT		
NbchIP-qPCR-F	TGAGGGCCGATTCCAAAATCA	Efficiency: 98.82%	
NbchIP-qPCR-R	CGCCTGAACATTTTGTCACGT		
NbSAHH1α-qPCR-F	GGTCTCATGGCTTGTCGTACTG	Efficiency: 93.10%	
NbSAHH1α-qPCR-R	TTCAGCACCCAAAGCAGTAAGG		
Actin-145F*	AAAGACCAGCTCATCCGTGGAGAA	Efficiency: 110.38%	
Actin-145R *	TGTGGTTTCATGAATGCCAGCAGC		
RSV-9200F*	GAGCAATGCTTACGGATGGC	Efficiency: 93.65%	
RSV-9350R*	GATCATAGCATGGCCAGTGC		

Note:The primers indicated with asterisks and wells were designed in previous publications[10,24,57].

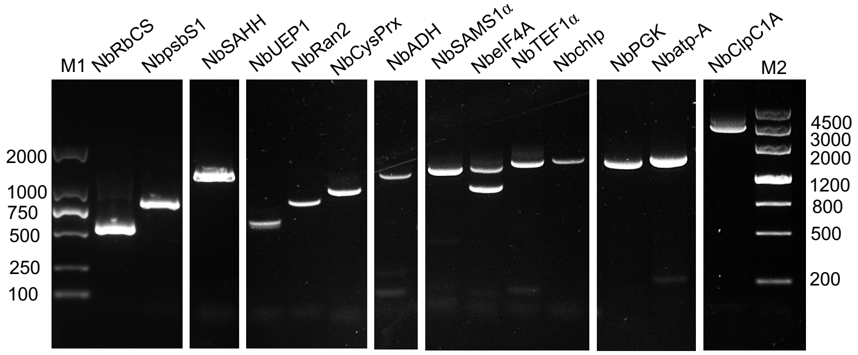
M1
NbRbCS
NbpsbS1
NbSAHH
NbUEP1
NbRan2
NbCysPrx
NbADH
NbSAMS1α
NbeIF4A
NbTEF1α
Nbchlp
NbPGK
Nbatp-A
NbClpC1A
M2
2000
1000
750
500
250
100
4500
3000
2000
1200
800
500
200

Figure S1

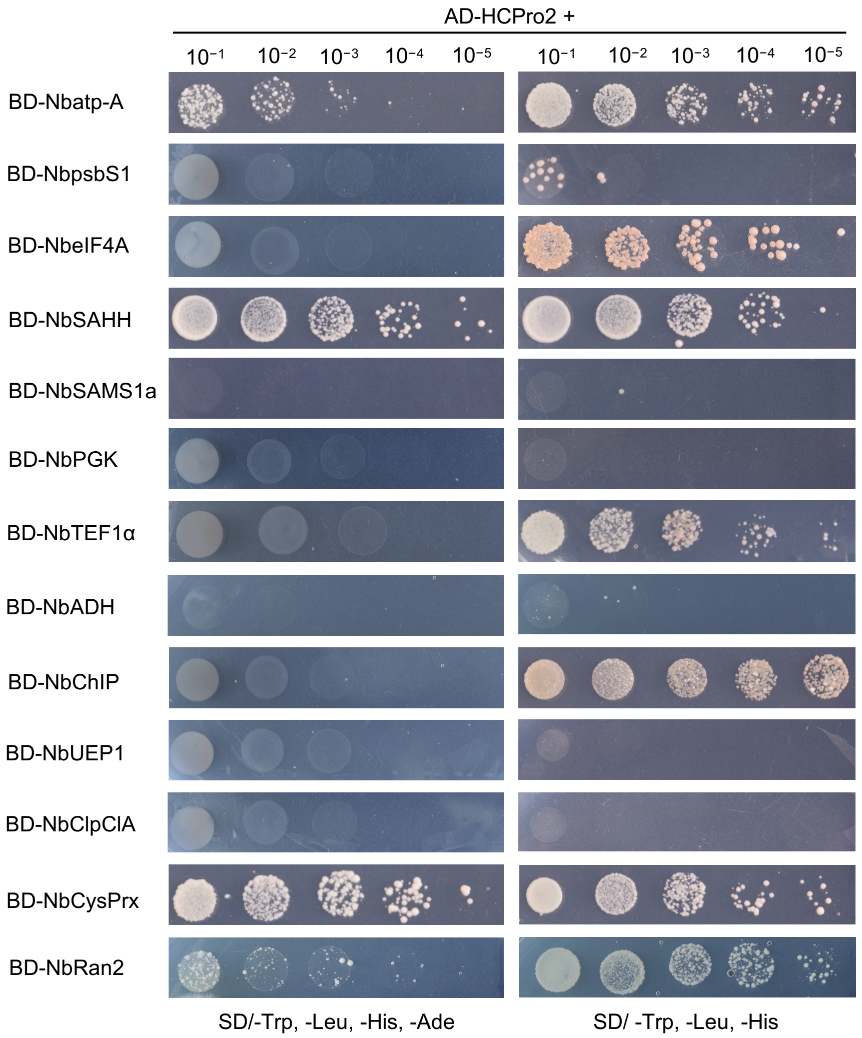
AD-HCPro2 +
10−1
10−2
10−3
10−4
10−5
10−1
10−2
10−3
10−4
10−5
BD-Nbatp-A
BD-NbpsbS1
BD-NbeIF4A
BD-NbSAHH
BD-NbSAMS1a
BD-NbPGK
BD-NbTEF1α
BD-NbADH
BD-NbChIP
BD-NbUEP1
BD-NbClpClA
BD-NbCysPrx
BD-NbRan2
SD/-Trp, -Leu, -His, -Ade
SD/ -Trp, -Leu, -His

Figure S2

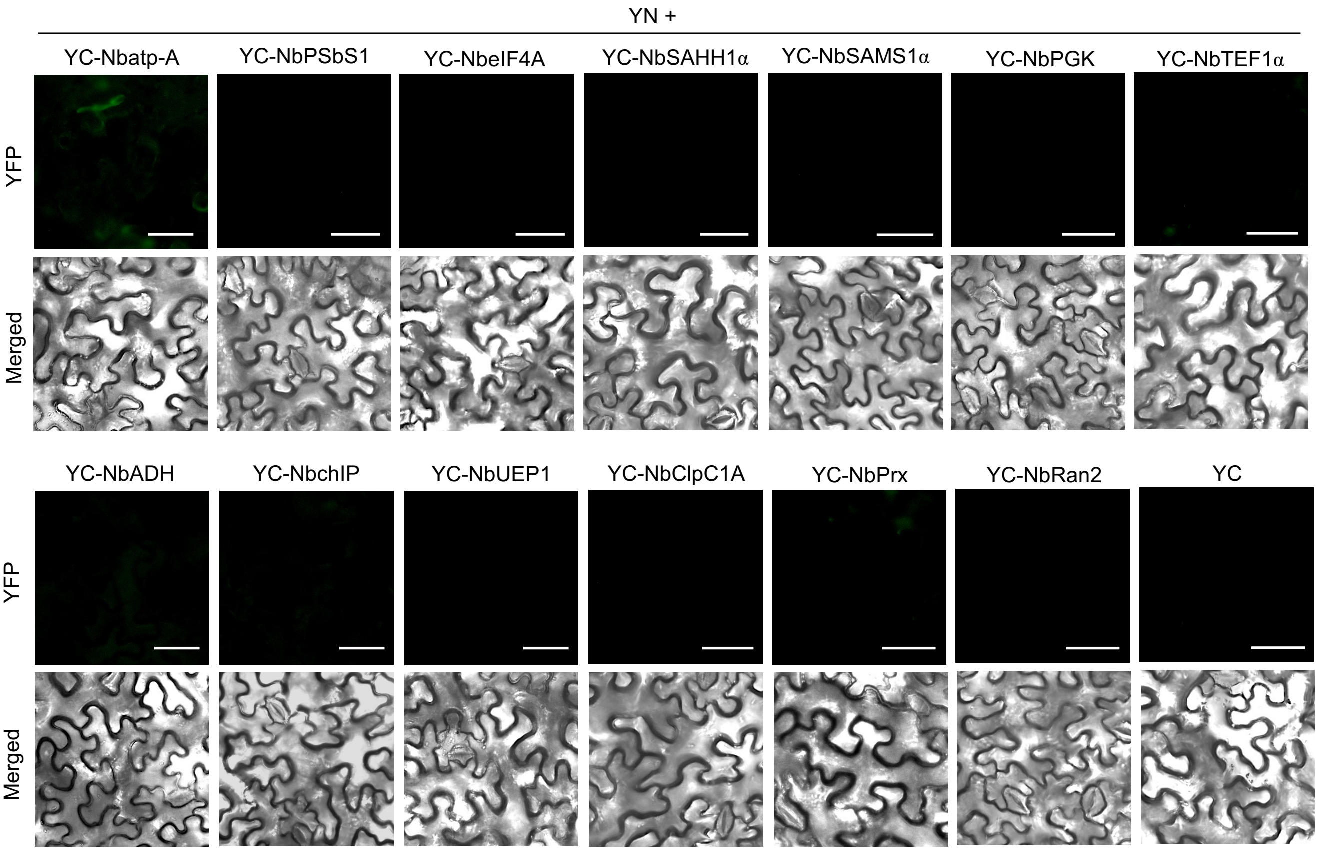
YN +
YC-Nbatp-A
YC-NbPSbS1
YC-NbeIF4A
YC-NbSAHH1α
YC-NbSAMS1α
YC-NbPGK
YC-NbTEF1α
YFP
Merged
YC-NbADH
YC-NbchIP
YC-NbUEP1
YC-NbClpC1A
YC-NbPrx
YC-NbRan2
YC
YFP
Merged

Figure S3

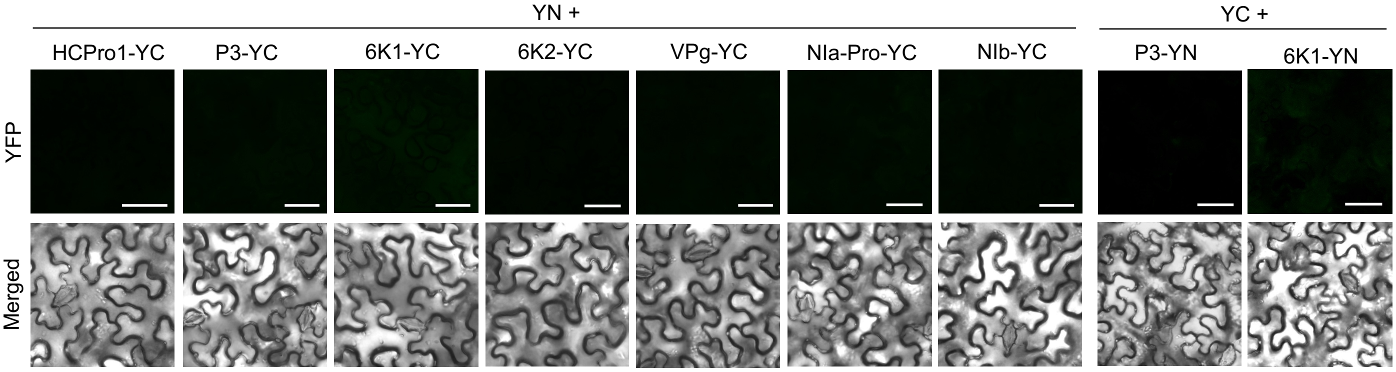
YN +
YC +
HCPro1-YC
P3-YC
6K1-YC
6K2-YC
VPg-YC
NIa-Pro-YC
NIb-YC
P3-YN
6K1-YN
YFP
Merged

Figure S4
